# The mnemonic potency of functional facts

**DOI:** 10.3758/s13423-024-02617-x

**Published:** 2024-12-20

**Authors:** Stuart Wilson

**Affiliations:** Queen Margaret University, Edinburgh, Scotland EH21 6UU UK

**Keywords:** Functional memory, Semantic memory, Adaptive memory, Cultural memory; Mnemonics

## Abstract

Learning and remembering what things are used for is a capacity that is central to successfully living in any human culture. The current paper investigates whether functional facts (information about what an object is used for) are remembered more efficiently compared with nonfunctional facts. Experiment 1 presented participants with images of functionally ambiguous objects associated with a (made-up) name and a (made-up) fact that could relate either to the object’s function or to something nonfunctional. Results show that recall of object names did not depend on whether they were associated with a functional or nonfunctional fact, while recall of the functional facts was significantly better than the nonfunctional facts. The second experiment replicated this main effect and further found that functional facts are remembered more efficiently after they have been associated with confirmatory (as opposed to disconfirmatory) feedback. It is suggested that semantic information is not unitary, and that one way of categorising semantic information is in terms of its adaptive relevance. Potential mechanisms are proposed and discussed, along with suggestions for future research.

## Introduction

Humans are not the only tool-using species (see, e.g., Seed & Byrne, 2010), but the extent of our tool use and the complex nature of how we use and manipulate objects to meet our needs puts us far beyond any other creature. This has attracted interest from cognitive scientists, who endeavour to explain how these capacities can be explained at a neurocognitive level (e.g., Vaesen, 2012). A cognitive capacity that humans seem to particularly excel at is being able to think about the *functions* of objects. Humans are exceptionally good at conceptualising an object as being “for” a particular purpose, to the extent that, once we have established in our minds what the function of an object is, we often have difficulty thinking of it in any other way, as evidenced by the well-known phenomena of “functional fixedness” (Duncker, 1945). Indeed, studies have shown that being informed just once about an object’s function is sufficient to hinder any deviation from this conceptualisation (Defeyter & German, 2003). Information about function, it seems, is cognitively potent, and many theorists have suggested the existence of a conceptual system that is dedicated to the organisation and storage of functional information (Vaesen, 2012).

The link between thinking about function and memory has not received a great deal of attention beyond a small number of studies. Schacter and Cooper (1993) had participants think about possible functions of novel objects, finding that subsequent recognition memory was improved compared with when the objects were encoded in relation to structural information (e.g., whether the objects were facing left or right). More recently, functional encoding has been proposed as a potential proximate mechanism to explain the so-called survival processing effect (see Bell et al., 2015; Kroneisen et al., 2021; Wilson, 2016), a mnemonic phenomenon in which recall is enhanced when stimulus items are processed with reference to a grasslands survival schema (Nairne et al., 2007).

The current paper’s focus is on functional knowledge itself, and whether this kind of information is prioritised by mnemonic systems. There is a growing literature on how (and why) mnemonic systems prioritise information. For example, investigating whether “valued” stimuli are prioritised in memory, Murphy and Knowlton (2022) assigned arbitrary scores to stimulus items, finding an asymmetry in recall that depended on whether participants’ goals were framed in terms of gains (e.g., “do your best to maximise your score”) rather than losses (e.g., “do your best to minimise your losses”; see Knowlton & Castel, 2021, for a review of value-based learning). Additionally, a broad range of studies have established a family of “self-reference” effects that make certain things more memorable when they are attached to our self-concept in some way (see Klein, 2012, for a review). Other research has attempted to investigate the nature of mnemonic priorities by deriving predictions from evolutionary theory. For example, Kroneisen (2018) found that information about another individual’s past behaviour (and how relevant it is to you) can influence how memory operates in relation to that person, a finding that supports the idea that memory systems in species that engage in frequent reciprocal cooperative interactions will be sensitive to cues relating to the trustworthiness of potential exchange partners.

The current approach is derived from the functionalist agenda in memory research (Nairne & Pandeirada, 2010). As Wilson et al. (2011) point out, not all stimuli in the world are equal in terms of their fitness relevance, and so we might expect that certain forms of information are privileged in terms of how they are processed. Why might we expect information about the function of objects to have fitness relevance and be prioritised in memory? Learning and storing information relating to object function is hugely important from a socio-cultural standpoint. Being able to think about the function of objects is central to most human cultures. Human life is based around using a vast array of objects, and individuals are required to learn an enormous number of functional facts over a lifetime. This kind of learning is arguably a fundamental feature of human life and could be considered as being of acute importance given that survival in cultural groups depends upon an individual becoming familiar with the tools that exist in the shared environment. Failure to efficiently learn about the functions of the artefacts in one’s cultural environment would put an individual at a severe disadvantage. To the extent that humans can be said to have a “capacity” for culture, this capacity must in part consist in cognitive skills that allow for the efficient detection, encoding and retrieval of information relating to the function of objects. Having an internal “database” of functional information also allows individuals to effectively communicate with each other about useful artefacts, facilitating the “ratcheting” effect that drives cultural evolution by allowing innovations to be built upon existing knowledge (Tennie et al., 2009). Being able to efficiently process, store, and recall information about function is arguably more important than performing identical cognitive tasks on other (nonfunctional) object-related information. Functional facts have more adaptive value than other kinds of facts, including (arguably) what an object is called. This line of reasoning suggests that learning about the functions of objects should be particularly privileged in a way that learning about other object-related facts may not be. It might further be argued that information about function should also be more sensitive to feedback compared with other forms of semantic information. This is because the adaptive consequences of being wrong about functional information are greater than the consequences of being wrong about any other form of semantic information that could be attached to objects (e.g., names).

The current studies aim to investigate the link between functional information and memory. Given the socio-cultural importance of learning functional information and the adaptive benefits that prioritising such information might have, the expectation is that this form of semantic information will be prioritised mnemonically in comparison with semantic information that is not functional in nature.

## Experiment 1

The first study presented participants with functionally ambiguous objects, along with a made-up name and a piece of information that could either be about the function of the object (a “functional-fact”) or about something nonfunctional.

Predictions were as follows:

1. There will be significantly elevated recall for “functional facts” about the objects compared with “nonfunctional facts”, and
2. there will be no significant difference in the recall rates for the names of the objects associated with “functional facts” compared with “nonfunctional facts” (because the names of objects convey no information about function).

Institutional ethical approval was granted for both Experiment 1 and 2, which were conducted in accordance with all ethical standards as set out by the British Psychological Association.

Sample size was set at 50 as a sensitivity analysis revealed that it was possible to detect an effect of *d*_z_ = 0.42 for the comparison between functional and nonfunctional facts with a statistical power of 0.90 in a one-tailed test.

### Method

#### Participants and design

Participants were undergraduate students participating for course credit. Fifty participants took part (mean age = 22.6 years, 21 men, 29 women) in a repeated-measures study in which recall of the name and the information associated with functionally ambiguous objects was measured across two conditions: functional information and nonfunctional information.

#### Materials

**Fig. 1:**
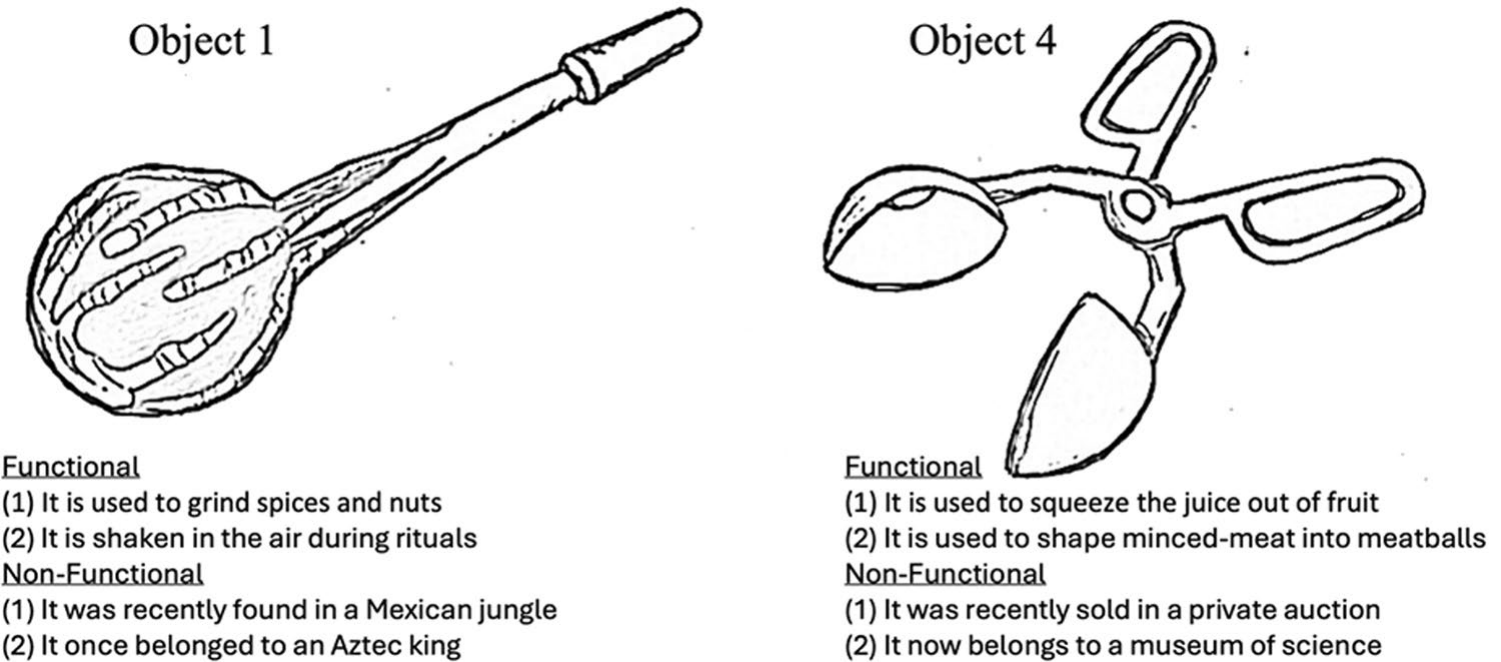
Illustrations of two objects and accompanying facts. Note that due to copyright reasons the original photographs used in the study cannot be included. The images below are an artist’s reproduction of the original images, which were in full colour

Forty-five images of functionally ambiguous/unusual objects were collected from various websites and internet message boards that specialise in such objects. The sourcing of the images began with searches on various image search engines for “unusual” “unknown” and “ambiguous” objects. This yielded some usable images and also provided links to online locations where such objects are posted and discussed.^1^ These locations were also searched for suitable images until enough had been found. For an image to be considered for inclusion it had to depict an object on its own (i.e., the image could not depict an object in human hands or alongside other objects). Objects selected were all an appropriate size to be held in the hands and manipulated/used. Suitable objects were those for which the function was not obvious or was ambiguous. Some objects resembled known objects but did not obviously perform the same function as the object they resembled. For example, Object 4 resembled a pair of scissors in that it had handles with looped slots through which a thumb and fingers could be placed. However, rather than cutting blades, the two extensions contained hemispherical cups that formed a full sphere when the “blades” were brought fully together (see Fig. 1). Images were edited to be approximately the same size when presented in the centre of the screen. Although images varied in resolutions, no image was included that did not depict the object in a way that its potential functions could be adequately discerned.

Because the author does not own the copyright to any of the images, they cannot be included in the online materials. See Fig. 1 for an artist’s reproduction of two of the images. Similar images can be found online and anyone replicating this work is encouraged to use independently sourced images.

Names associated with each image were two syllable nonsense words that did not obviously resemble any known names for objects or tools (e.g., Loftog; see the Appendix Table 1 for a list of the names used). Functional and nonfunctional information for each object was created by the experimenter and were not intentionally related to the actual function of the objects (which were unknown anyway). Four facts were created for each object, two functional and two nonfunctional. Each “fact” had to be conceivable when associated with the object (i.e., participants had to view the provided functional information as something that would be possible given the object that it was associated with—e.g., a functional fact that was associated with a hook-like object was “this object was used to weigh bales of hay” and not “this object is used to measure angles in buildings”, which would not be conceivable given the features of the hook-like object).

Functional facts were chosen to convey some way in which the object could be used. Nonfunctional facts were chosen such that they conveyed a generally interesting piece of information about the object but did not include details about its function. These facts tended to be related to things like where the object originates from, where it currently resides, who once owned it, and so forth. For example, the previously mentioned Object 4 (see Fig. 1) was assigned the following functional facts: (a) “It is used to squeeze juice out of fruit”; (b) “It is used to shape minced meat into meatballs”; and the following nonfunctional facts: (c) “It was recently sold at a private auction”; (d) “It now belongs to a museum of science”. Only one of these facts would be presented on any given trial; see the Appendix Table 1 for a full list of the functional and nonfunctional facts.

#### Procedure

Participants read an information sheet and signed a consent form. After providing basic demographic details, participants first took part in a series of practice trials that replicated the real trials described below. These practice trials used stimuli that were not used in the main trials.

At the start of the session, participants were told that they were about to see a series of unusual objects. They were informed that the first part of the experiment involved them answering two questions for each object: (1) “Do you recognise this object?” and (2) “Do you know what this object is used for?” and that both questions would be answered on a 7-point scale.^2^ These measures were taken so we could assess how “ambiguous” the stimuli were in our sample. Participants were further told that, after they had rated the objects, they would see each one again. On this presentation, each object would be accompanied by a name and a piece of information about the object. They were asked to pay close attention to this information because their memory would be tested for it later in the study. On each of the 44 encoding trials, the object and associated information were presented on-screen for 7 s, with the image of the object appearing in the centre of the screen. Directly under the object, the name was presented in the following way: “This is a _____”, and directly below this was the functional or nonfunctional fact. For each participant, half of the objects presented were associated with functional facts, and the other half associated with non-functional facts. Order of presentation was randomised. Which fact was chosen for each object (from the two available in each information condition) was also randomised.

After viewing all objects and associated names/facts, participants proceeded to the recall stage after a 3-min distraction task that involved counting backwards from a given number when cued. For the name recall, each object was presented alongside two names, one of which was the name that was presented in the first part of the experiment, the other being a name that had not been used at any point previously. For the fact recall, the object was shown along with an instruction to type into a textbox the fact that had been associated with the object in the earlier phase. After completing these recall tasks for all objects, participants were thanked and debriefed.

### Results and discussion

Firstly, the scores from the rating tasks relating to recognition of objects and knowledge of object function were calculated. The average recognition score for the objects used in the study was 2.55 (*SD* = 0.93). The average score for the recognition of function was 2.24 (*SD* = 0.76). Given that the highest score on these measures was 7, these data show that the objects were, as intended, generally unknown to the sample.

Responses in the information recall task were given to two independent raters who were blind to the hypotheses. Interrater reliability was high (Cohen’s Kappa = 0.85). On trials where the two raters disagreed, a third rater was used and a majority-vote method provided the final decisions.

When asked to decide between two options which name had been associated with each object during the encoding phase, the mean number of correct responses for objects that had been previously associated with functional facts was 13.22 (*SD* = 2.71) and was 13.30 (*SD* = 2.18) for objects that had been associated with nonfunctional facts. This difference was not statistically significant: *t*(49) = 0.18, *p* = 0.86 (two-tailed^3^), Cohen’s *d* = 0.03.

When asked to recall the fact that had been associated with each object, the mean recall score for functional facts was 13.32 (*SD* = 5.01); the mean recall score for nonfunctional facts was 9.74 (*SD* = 4.62). This difference is statistically significant: *t*(49) = 7.94, *p* < 0.001 (one-tailed) Cohen’s *d* = 1.12.

These results support the hypothesis that functional facts would be better remembered than nonfunctional facts. As expected, recall of the names of the objects did not differ according to whether the object was associated with a functional fact compared with a nonfunctional fact. Functional information, it seems, is particularly memorable. Experiment 2 was conducted as a way of further investigating this effect.

## Experiment 2

The second experiment aimed to further explore the mnemonic potency of information about the function of objects. As shown by the results of Experiment 1, when learning new facts about an unfamiliar object, we are more efficient when the information is of a specific semantic form—that of information about the object’s function. Whereas Experiment 1 was concerned with asking about the potential mnemonic advantages of the functional information itself, Experiment 2 additionally asks about whether receiving feedback (either confirmatory or disconfirmatory) on one’s initial judgment of an item’s function influences subsequent recall of that functional information.

There are multiple ways to conceptualise the role of feedback in this context, making it hard to make specific predictions. Intuitively, we might expect that information confirming an item’s true function would be most likely to be recalled because that information has most utility in terms of interacting with the object appropriately in the future. Confirmatory feedback has also been associated with dopamine release (Doll et al., 2011), which may have implications for subsequent recall performance (Shohamy & Adcock, 2010). Receiving confirmatory feedback could also trigger a “truth-effect”, such that the information increases in cognitive fluency and is thus remembered more efficiently (see Dechêne et al., 2010, for a review and meta-analysis).

It could also be argued, however, that instances where we are mistaken about functional information should be most memorable. Some theorists have emphasised that an adaptive and optimised cognitive system should be flexible and thus should prioritise information that violates expectancies (see, e.g., Bell et al., 2012). If we think an object is for one thing but are subsequently informed that it is actually for something else (unexpected disconfirmatory feedback), this could be especially important to remember, because using objects in the wrong way can be harmful in both a physical and a social sense. Remembering functional information that is contrary to one’s initial estimates might be important given the potential costs of continuing to misconstrue the function of the object into the future.

Additionally, the ongoing debate over how “truth value” is represented in memory (Gilbert, 1991) is also relevant when considering the role of feedback. Briefly, the Spinozan model holds that new information is automatically believed to be true at encoding, with further processing only required to update this and tag the information as false. The Cartesian model suggests that new information is initially neutrally represented, with truth value established later, when a true or false tag is attached. If the Spinozan model is correct, information associated with confirmatory feedback will receive less processing than information associated with disconfirmatory feedback. If the Cartesian model is correct, then both forms of feedback will be associated with similar levels of cognitive effort (see Bernhard et al., 2023; Nadarevic & Erdfelder, 2019).

All these scenarios have implications for how to conceptualise the effects of feedback in the current task. As such, no directional predictions were made for the role of feedback on recall of functional facts. Because functional information is adaptively more important than nonfunctional information, it is expected that feedback has more of an influence on the former.

Predictions were as follows:

1. Functional facts will be recalled more efficiently than nonfunctional facts (replicating the findings of Experiment 1).
2. Feedback will have more of an influence on recall of functional facts compared with nonfunctional facts (i.e., there will be an interaction between fact type and feedback).

No specific predictions were made regarding the type of feedback and its effect on recall.

A power analysis was conducted using the effect size from the previous experiment (*d*_z_ = 1.12). This suggested that a sample size of 11 would be sufficient to detect the previously detected difference between functional and nonfunctional facts with a statistical power of over 0.90 in a one-tailed test. As the effect size for the second comparison (the effect of feedback) was unknown, another analysis was conducted assuming a small effect size (entered as half the size of the known effect in the other condition: *d*_z_ = 0.56). This suggested that a sample size of 44 was required for statistical power of 0.95 in a two-tailed test. Sample size was thus set at around 50.

### Method

#### Participants and design

Fifty-one participants (mean age = 27.01 years, 16 men, 35 women) took part in a repeated-measures design consisting of 2 (facts: functional vs. nonfunctional) × 2 (feedback: confirmatory vs. disconfirmatory) factors. Participants were from the same sampling pool as in the previous experiment and none had taken part in Experiment 1.

#### Materials and procedure

All materials were identical to those used in the previous experiment. The first stages of Experiment 2 were identical to the previous experiment. After consenting and taking part in practice trials, participants viewed and rated 44 objects for recognition and function. The encoding phase differed from the previous experiment. During this phase, each object was associated with two made-up “facts” about the object. Half of the trials involved pairs of functional facts, and the other half involved pairs of nonfunctional facts. On each trial, participants were asked to choose which fact they thought was correct and were provided with immediate feedback as to the (pseudo) correctness of their response. Which response was deemed “correct” was randomly allocated and was not associated with any known information about any of the objects. After completing the encoding phase, participants completed the same 3-min distractor task described in Experiment 1, and then moved on to the recall phase, in which they were presented again with the 44 objects they had seen during the encoding phase and were asked to enter into a text box the fact that they had previously learned about the object. After doing this for all objects, participants were thanked and debriefed.

### Results and discussion

Responses from the recall task were given to two independent raters who were blind to the hypotheses. They displayed high interrater reliability (Cohen’s Kappa = 0.82). As in the previous study, disagreements were given to a third rater and a majority-vote method decided disagreements.

As in the previous experiment, descriptive statistics for the recognition ratings were calculated. When asked to rate general recognition of the objects, the average recognition rating was 2.42 (*SD* = 0.76). When asked to rate recognition of the function of the objects, the average recognition rating was 2.22 (*SD* = 0.68). Again, these descriptive statistics demonstrate that the stimuli were not generally recognised by the sample.

Due to the randomisation process, participants did not all view the same number of trials with confirmatory and disconfirmatory feedback,^4^ so the analyses below are conducted on proportions of correct responses rather than absolute values,^5^

For items paired with functional information, the proportion of items correctly recalled when participants were given confirmatory feedback was 0.64 (*SD* = 0.23); the proportion when participants were given disconfirmatory feedback was 0.56 (*SD* = 0.26). For items paired with nonfunctional information, the proportion correctly recalled when participants were given confirmatory feedback was 0.45 (*SD* = 0.23); the proportion when participants were given disconfirmatory feedback was 0.48 (*SD* = 0.22).

**Fig. 2:**
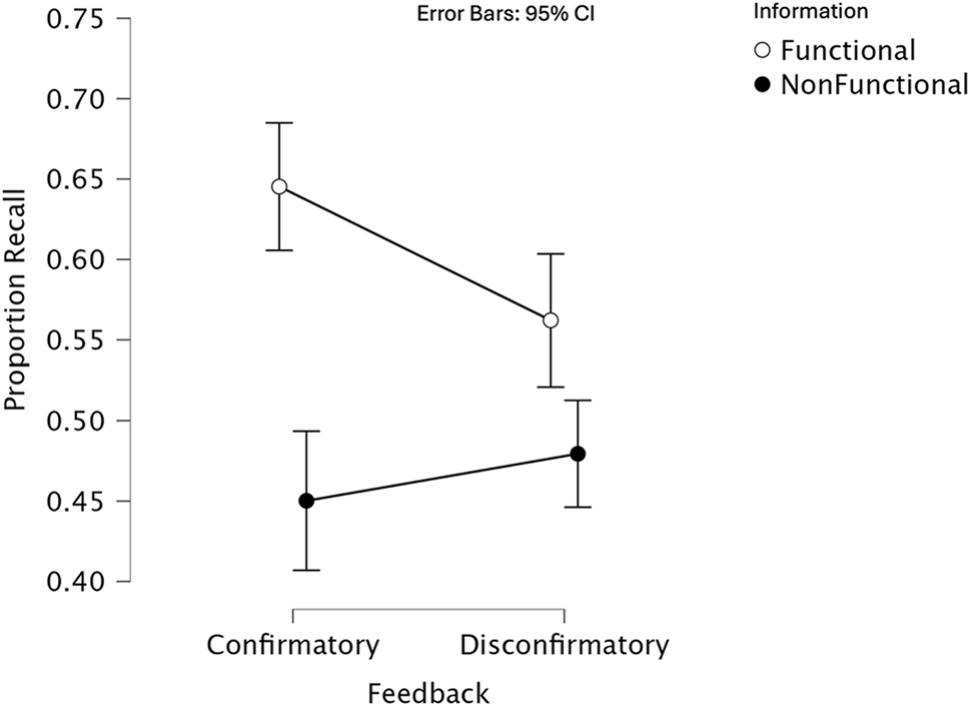
Recall rates for two information types when provided with confirmatory and disconfirmatory feedback

A 2 (information: function vs. nonfunction) × 2 (feedback: confirmatory vs. disconfirmatory) repeated-measures analysis of variance (ANOVA) was conducted. There was no main effect for feedback type, *F*(1,50) = 1.49, *p* = 0.23, η_p_^2^ = 0.03. There was a main effect for information type, *F*(1,50) = 31.77, *p* < 0.001, η_p_^2^ = 0.39, confirming the first hypothesis and replicating the effect found in Experiment 1. There was also a significant interaction, *F*(1,50) = 7.11, *p* = 0.01, η_p_^2^ = 0.12. The nature of this interaction is explored below and shown in Fig. 2.

All post hoc simple effects analyses employ two-tailed statistics. Comparing functional and nonfunctional information that was associated with confirmatory feedback (“correct”) during the encoding phase found a significant difference in subsequent recall, with functional information being recalled more efficiently, *t*(50) = 5.62, *p* < 0.001, Cohen’s *d* = 0.79. Comparing functional and nonfunctional information associated with disconfirmatory feedback (“incorrect”) during encoding also revealed a significant difference in subsequent recall, with functional information being recalled more efficiently, *t*(50) = 2.76, *p* = 0.008, Cohen’s *d* = 0.39. The difference in these effect sizes is one source of the observed interaction effect.

Recall on items associated with functional information was compared across trials on which participants were given confirmatory feedback and trials on which participants were given disconfirmatory feedback, revealing that there was a statistically significant difference with the confirmatory feedback resulting in better recall, *t*(50) = 2.62, *p* = 0.01, Cohen’s *d* = 0.37.

When the recall of items associated with nonfunctional information was compared in the same way, no statistically significant difference was found between the trials on which confirmatory feedback was provided compared with trials in which disconfirmatory feedback was provided, *t*(50) = 0.99, *p* = 0.33, Cohen’s *d* = 0.14. This further reveals the source of the interaction (see Fig. 2).

The results from Experiment 2 show that functional information is recalled especially well after positive feedback. As can be seen in Fig. 2, the pattern for nonfunctional information across the feedback conditions stands in contrast to that for the functional information, suggesting once again that functional information is processed differently compared with nonfunctional information, with recall of the former appearing to be more susceptible to confirmatory feedback. This suggests that there are different cognitive consequences when being told that we are correct when the information under consideration is functional in nature compared with when it is nonfunctional information. Again, this highlights the unique nature of functional information.

#### Post hoc* analyses*

To further investigate potential mechanisms associated with the observed recall advantage for functional facts, post hoc measures of plausibility, concreteness, and imageability were collected for all object/info pairings (see the Appendix Table 1). Statistically significant differences between functional and nonfunctional facts were found for all three measures.^6^ To explore the degree to which these factors were implicated in the functional advantage, the following analyses were conducted on the recall data from Experiment 1 using the stimulus items as the units of analysis. For each measure (plausibility, concreteness, and imageability) 10 object–info pairings from each condition (functional facts/nonfunctional facts) were selected that had average ratings in the middle of the response scale (i.e., around 3). Items chosen on plausibility had average ratings ranging from 3.00 to 3.20.^7^ When this matched subset was compared on recall, the functional advantage persisted, *M*_Functional_ = 0.66, *SD* = 0.19; *M*_Nonfunctional_ = 0.50, *SD* = 0.23, *t*(18) = 1.68, *p* = 0.055, Cohen’s *d* = 0.75, one-tailed. Although this falls just short of significance, the effect size indicates a medium/large effect, suggesting that the recall advantage associated with functional facts cannot be fully explained by differences in how plausible the facts were when associated with the objects. A similar analysis was conducted on 10 object–info pairings from each condition matched on concreteness (averaged ratings ranged from 3.00 to 3.17).^8^ Again, this matched subset showed a significant recall advantage for the functional facts, *M*_Functional_ = 0.60, *SD* = 0.23; *M*_Nonfunctional_ = 0.39, *SD* = 0.24, *t*(18) = 1.96, *p* = 0.03, Cohen’s *d* = 0.88, one-tailed. This result suggests that matching items for concreteness does not diminish the effect. Finally, the same procedure was applied to items matched on imageability (averaged ratings ranged from 3.00 to 3.20).^9^ Although the functional facts were better remembered than the nonfunctional facts for this subset, the difference did not approach significance, *M*_Functional_ = 0.57, *SD* = 0.24; *M*_Nonfunctional_ = 0.47, *SD* = 0.23, *t*(18) = 0.95, *p* = 0.18, Cohen’s *d* = 0.43, one-tailed. This suggests that the capacity to form images relating to the items and their suggested function may be implicated in the observed recall advantage.

## General discussion

Two experiments investigated the mnemonic potency of functional information by utilising functionally ambiguous objects and pairing each object with either a functional or nonfunctional fact. The first experiment established that functional facts are better remembered than nonfunctional facts, and that recall of item names is not influenced by type of fact attached to the objects. Experiment 2 replicated the main finding of the previous experiment and also revealed that functional information is especially sensitive to confirmatory feedback.

Post hoc analyses on matched subgroups suggested that the recall advantage did not seem to be related to concreteness or plausibility but may be related to how easy the object–info pairings were to imagine. It would be of interest for future studies to investigate this further. For example, is the effect of imageability in these tasks related to self-reference effects? Do higher imageability scores relate to the ease with which the object–info pairings can be visualised in terms of self-related actions (whether imagining oneself using the object, or imagining the object used on oneself), and is this related to subsequent recall? The possibility that the encoding task produces a larger self-reference effect for functional information compared with nonfunctional information is a question that requires more empirical work. Thinking about the function of an object usually involves imagining engaging with it personally (e.g., performing the function). Encoding the nonfunctional facts related to the objects is unlikely to involve similar levels of imagined personal engagement (although this is something that should be empirically established in future studies).

There are other potential mechanisms worth considering, such as the influence of relevance/value. It seems self-evident that facts about an object’s function will be seen as more important than nonfunctional facts, so the observed pattern of results could also be a consequence of this prioritisation (and the question of where this relevance comes from and how it interacts with feedback can be a focus for future work). Any difference in the relevance or value placed on the two forms of information could also mean that we are also seeing an effect of motivation, as people may be intrinsically more motivated to think about the potential uses for ambiguous objects than they are to think about nonfunctional facts (and the question of how motivation interacts with feedback could be another focus for future work).

In suggesting possible mechanisms for the observed recall advantage, it is important to note that identifying mechanistic explanations does not necessarily invalidate the suggestion that the mnemonic advantage serves a specific function, as functional and mechanistic explanations can complement each other and should not be considered exclusive (see Shapiro, 2017). If it is indeed adaptive to prioritise functional information in memory, then this, for example, may be achieved by making functional information easier to imagine. Furthermore, the suggested mechanisms cannot fully explain the pattern of results observed in Experiment 2. The finding that the type of feedback interacts with the nature of the information suggests a more complex picture than can be painted by appealing to mnemonic mechanisms, and this should also be a focus of future work.

Finally, there is the possibility that imagining using objects is simply a more cognitively demanding task that combines multiple mechanisms. In comparison to the processing required for the nonfunctional facts, imagining how an object might be used in the way described by the presented information involves a series of processes that make sense of the object’s physical constitution; the mechanics of how the object can be manipulated; a representation of the end-goal; and a personal involvement with the object that one is imagining the function of. This is arguably a much more involved encoding process than is needed for imagining an object being held in a museum (to use an example of a nonfunctional fact from the current stimuli). Because the post hoc ratings would have required similar processing, it is possible that the effects of these multiple mechanisms influenced the ratings of concreteness, plausibility, and imageability for the functional facts, inflating their ratings compared with the nonfunctional facts. Again, these suggestions should be empirically established in future work.

This research began with a question about whether different kinds of semantic information might be treated differently by memory systems depending on its adaptive utility. This way of formulating hypotheses is not common in mainstream memory research, but good arguments can be made about why this kind of thinking is appropriate in the field (Nairne & Pandeirada, 2010). It should be recognised that there has been no independent verification of the “adaptive value” ascribed to functional facts. It is possible that other factors underpin the mnemonic priority that functional facts seem to enjoy.

Future studies should aim to establish the effect further and investigate its boundary conditions and mechanisms. It would be especially informative to utilise object–info pairings that had been specifically matched in advance on mnemonically relevant variables.

The current data suggest that semantic information is not uniform in nature. The reasons why some semantic information is treated differently by mnemonic systems compared with other semantic information is something that needs to be incorporated into theoretical models of memory. It may (or may not) be the case that functional facts are better remembered because of the adaptive value that remembering such things had for our ancestors over evolutionary time.

## Data Availability

Data are openly available via the Open Science Framework (https://osf.io/a9qpv/).

## Appendix

**Table 1:** Functional/Nonfunctional facts and nonword names for 44 stimulus objects. Figures refer to means and standard deviations for plausibility (P), concreteness (C) and imageability (I)

	Functional Fact 1 (F1)	Functional Fact 2 (F2)	Nonfunctional Fact 1 (N1)	Nonfunctional Fact 2 (N2)	Name1*	Name2*
Object01	It is used to grind spices and nuts P = 3..71 (1.34) I = 3.83 (1.29) C = 3.91 (1.15)	It is shaken in the air during rituals P = 4.09 (1.07) I = 4.17 (0.95) C = 3.43 (1.36)	It was recently found in a Mexican jungle P = 2.77 (1.26) I = 2.43 (1.14) C = 2.54 (1.17)	It was once owned by an Aztec King P = 3.46 (1.27) I = 3.40 (1.22) C = 2.94 (1.35)	Linsog	Dimpot
Object02	It is used as a “clothes-peg” for securing heavy leather material P = 3.17 (1.42) I = 3.03 (1.38) C = 2.91 (1.34)	It is a spring-loaded trap for small and medium-sized animals P = 3.89 (1.08) I = 3.83 (1.18) C = 3.74 (1.22)	It is one of the first Iron-Age objects ever discovered P = 3.60 (1.26) I = 3.31 (1.23) C = 3.31 (1.13)	It was invented in Russia during the 10th Century P = 3.51 (1.22) I = 2.86 (1.22) C = 2.91 (1.27)	Flargut	Jaktu
Object03	It is used to get the lids off heavy wooden barrels *rating data not available due to a coding error	It is used to pull unwanted nails out of wooden surfaces P = 3.46 (1.24) I = 3.29 (1.27) C = 3.29 (1.49)	It is the only one known to have survived World War Two P = 2.89 (1.47) I = 2.26 (1.20) C = 2.34 (1.21)	It is the original version of a popular high-street brand P = 2.40 (1.14) I = 2.14 (1.14) C = 2.11 (1.11)	Sinfald	Loftog
Object04	It is used to squeeze juice out of fruit P = 3.66 (1.41) I = 3.77 (1.24) C = 3.40 (1.40)	It is used to shape minced-meat into meatballs P = 3.77 (1.21) I = 3.86 (1.17) C = 3.40 (1.40)	It was recently sold at a private auction P = 3.83 (1.04) I = 3.71 (1.27) C = 3.57 (1.14)	It now belongs in a museum of science P = 3.11 (1.47) I = 3.00 (1.43) C = 2.74 (1.44)	Urdall	Hantog
Object05	It is used to make simple skin tattoos P = 3.11 (1.37) I = 3.14 (1.38) C = 3.06 (1.33)	It is used to tenderise meat before cooking P = 3.51 (1.46) I = 3.83 (1.32) C = 3.63 (1.26)	It is the oldest one ever discovered P = 3.60 (1.26) I = 3.069 (1.25) C = 2.69 (1.23)	It is the smallest one ever discovered P = 3.06 (1.21) I = 2.63 (1.11) C = 2.29 (1.13)	Nomtaw	Lifwah
Object06	It is an example of an open-fire toaster P = 3.23 (1.48) I = 3.11 (1.60) C = 3.03 (1.36)	It is an early example of a heated seat P = 2.31 (1.32) I = 2.51 (1.44) C = 2.29 (1.38)	It was used as a prop in a Hollywood film P = 3.40 (1.24) I = 2.94 (1.33) C = 2.89 (1.16)	It belonged to a Sergeant of the British Army P = 2.94 (1.28) I = 2.80 (1.21) C = 2.49 (1.04)	Ornspog	Poksmee
Object07	It is used to massage and treat minor muscle injuries P = 3.14 (1.40) I = 3.37 (1.35) C = 3.06 (1.43)	It is used as a weapon when attached to a whip P 3.57 (1.40) I = 3.80 (1.30) C = 3.49 (1.34)	Is thought to have been made from an iron compound P = 4.00 (0.97) I = 3.43 (1.14) C = 3.17 (1.27)	It’s the only one of its kind that has ever been found P = 2.69 (1.35) I = 2.83 (1.25) C = 2.54 (1.12)	Rogflan	Zentaph
Object08	It is used by structural engineers to measure angles in buildings P = 4.14 (0.91) I = 4.09 (0.89) C = 3.80 (1.11)	It is used to measure distances between balls in games of bowls/boules P = 3.43 (1.12) I = 3.46 (1.22) C = 3.20 (1.43)	Was one of the earliest things owned by a famous Italian artist P = 3.34 (1.37) I = 2.54 (1.24) C = 2.69 (0.99)	It was one of the few objects rescued the Titanic that was never identified P = 1.83 (0.89) I = 1.89 (1.02) C = 2.06 (1.23)	Indayso	Brimtol
Object09	It is used to cut steel chains P = 3.14 (1.38) I = 3.14 (1.33) C = 3.14 (1.54)	It is used to connect wires together P = 3.49 (1.20) I = 3.26 (1.20) C = 3.06 (1.00)	It is much heavier than it looks P = 4.37 (0.73) I = 4.00 (1.11) C = 3.60 (1.38)	It is from the early 20th Century P = 3.66 (1.06) I = 3.29 (1.30) C = 3.00 (1.19)	Kamstap	Drovit
Object10	It was used to punish disobedient or disrespectful children P = 3.29 (1.45) I = 3.03 (1.46) C = 2.66 (1.37)	It was used to fan a person of high status P = 4.31 (0.76) I = 4.26 (0.78) C = 4.23 (0.69)	It is an early example of green leather P = 2.46 (1.29) I = 2.23 (1.17) C = 2.54 (1.31)	It is an early example of Egyptian craft P = 3.69 (1.13) I = 3.40 (1.22) C = 3.14 (1.17)	Farlac	Elbink
Object11	It is used to hold delicate objects while they are being painted P = 3.57 (1.20) I = 3.37 (1.19) C = 3.54 (1.09)	It is used to shape the growth of small Bonsai trees P = 2.86 (1.44) I = 2.63 (1.59) C = 2.89 (1.28)	It was recently given the status of “rare & interesting antique” P = 3.57 (1.17) I = 2.74 (1.04) C = 2.83 (1.15)	It was recently featured on a popular TV antiques show P = 4.03 (1.04) I = 3.77 (1.19) C = 3.17 (1.32)	Hingot	Markap
Object12	It is used to mark leather when laid flat on the ground P = 4.20 (0.90) I = 4.11 (0.99) C = 3.83 (1.18)	It is used to make springs by compressing pieces of wire P = 3.43 (1.07) I = 3.11 (1.28) C = 3.20 (1.26)	Played an important role in early Roman history P = 3.09 (1.12) I = 2.46 (1.20) C = 2.51 (1.25)	It was buried alongside a famous Roman Emperor P = 2.66 (1.16) I = 2.34 (1.21) C = 2.40 (1.29)	Quargen	Umpall
Object13	It is used to scrape excess cement from stone walls P = 3.63 (1.19) I = 3.40 (1.31) C = 3.49 (1.27)	It is used to gather piles of loose hay P = 2.77 (1.44) I = 2.46 (1.42) C = 3.03 (1.46)	Its date of origin is unknown to archaeologists P = 3.37 (1.37) I = 2.71 (1.45) C = 2.46 (1.38)	It was invented by a Chinese prince P = 2.66 (1.26) I = 2.46 (1.20) C = 2.40 (1.17)	Frintog	Amtrope
Object14	It is used as to cut down and move thick tree branches P = 3.29 (1.13) I = 3.20 (1.26) C = 3.11 (1.11)	It is used as a “multi-tool” for preparing fish for cooking P = 3.29 (1.20) I = 3.03 (1.27) C = 3.23 (1.33)	It was found in a cave in a remote part of Northern Europe P = 3.63 (1.33) I = 3.09 (1.34) C = 2.80 (1.26)	It was used by early human settlers in Northern Europe P = 3.40 (1.24) I = 2.97 (1.34) C = 2.69 (1.32)	Ghorpan	Luthak
Object15	It is used to stretch woollen carpets without damaging the material P = 3.29 (1.05) I = 3.11 (1.18) C = 3.20 (1.18)	It is used as a catapult to launch small objects at enemies P = 3.23 (1.35) I = 3.46 (1.27) C = 3.31 (1.32)	It has been used every single day since it was built P = 2.51 (1.25) I = 1.97 (0.86) C = 2.06 (1.14)	It was the first invention to be patented in the 20th Century P = 3.20 (1.18) I = 2.57 (1.24) C = 2.46 (1.24)	Chamor	Ernpok
Object16	It screws into trees to support a hammock P = 4.17 (1.01) I = 4.40 (0.74) C = 4.03 (0.98)	It is an early example of a cork-screw P = 4.03 (1.12) I = 4.23 (0.88) C = 3.69 (1.30)	It is the smallest one in a collection of ten P = 3.71 (1.05) I = 3.46 (1.27) C = 2.94 (1.33)	It was only ever owned by married women P = 1.83 (0.89) I = 1.63 (0.84) C = 1.80 (1.02)	Arnco	Simpat
Object17	It is used in the preparation of food P = 1.77 (0.91) I = 1.94 (1.16) C = 2.31 (1.11)	It is used in the preparation of jewellery P = 3.63 (1.21) I = 3.20 (1.32) C = 3.31 (1.25)	It has a solid gold attachment P = 2.77 (1.37) I = 2.94 (1.37) C = 2.89 (1.28)	It has a solid oak handle P = 4.00 (1.14) I = 3.63 (1.24) C = 3.66 (1.21)	Linsog	Remdip
Object18	“Sacred aromatic herbs” are burned in this object during religious rituals P = 3.51 (1.27) I = 3.43 (1.31) C = 3.54 (1.34)	Freshly picked tobacco leaves are mixed and blended in this object P = 3.66 (1.16) I = 3.34 (1.24) C = 3.20 (1.28)	This object has been found at Prehistoric sites all over Europe P = 3.17 (1.15) I = 2.66 (1.03) C = 2.89 (1.18)	This object is a family heirloom thought to be worth thousands P = 2.49 (1.17) I = 2.14 (1.19) C = 2.23 (1.14)	Krandat	Vorbit
Object19	It is used to hold metal swords whilst sharpening the blade P = 3.20 (1.49) I = 3.17 (1.38) C = 3.06 (1.39)	It is used to hold fishing rods while the fisherman sleeps P = 2.14 (1.09) I = 2.31 (1.18) C = 2.31 (1.30)	It was one of the first objects to contain an internal spring P = 3.60 (1.01) I = 3.54 (1.09) C = 3.43 (1.17)	It was first used in factories making Ford motor cars P = 3.57 (1.22) I = 3.20 (1.23) C = 3.29 (1.32)	Millok	Bindo
Object20	It is used to separate thick metal chains that have become entangled P = 2.20 (1.26) I = 1.77 (1.00) C = 2.29 (1.36)	It is used to smooth down the material in thatched roofs P = 3.11 (1.37) I = 3.00 (1.31) C = 3.14 (1.33)	It is made of the heaviest metal available in the country P = 3.03 (1.27) I = 2.74 (1.22) C = 2.69 (1.25)	This is the only one ever made in this red colour P = 2.63 (1.26) I = 2.74 (1.22) C = 2.40 (1.38)	Bardee	Wolpit
Object21	Heavy objects are hung in a bag on the hook, swung around the head and thrown P = 2.66 (1.35) I = 2.71 (1.27) C = 2.74 (1.27)	It is attached to the back of a horse-drawn carriage allowing children to “surf” behind P = 2.17 (1.18) I = 2.43 (1.29) C = 2.20 (1.41)	This object is thought to have been invented at around the same time as the wheel P = 2.89 (1.45) I = 2.43 (1.29) C = 2.40 (1.31)	This object is one of the earliest known examples of a children’s toy P = 1.74 (1.12) I = 1.69 (1.05) C = 1.91 (1.20)	Oshkard	Irbiff
Object22	This object was used to weigh bales of hay at farms P = 3.66 (1.14) I = 3.40 (1.19) C = 3.51 (1.12)	This object was used to hang carcasses of animals at farms P = 3.63 (1.24) I = 3.37 (1.44) C = 3.57 (1.22)	It was once the most expensive item owned by farmers P = 2.89 (1.21) I = 2.60 (1.12) C = 2.26 (1.20)	It was once against the law not to keep these on farms P = 2.91 (1.04) I = 2.49 (1.07) C = 2.40 (1.19)	Whisken	Poksmee
Object23	It is used to inspect small pieces of hot metal P = 3.37 (1.17) I = 3.11 (1.32) C = 3.14 (1.19)	It is used to stamp small pieces of jewellery P = 3.91 (1.15) I = 3.63 (1.29) C = 3.34 (1.33)	It is constructed from solid silver P = 3.03 (1.34) I = 2.80 (1.32) C = 3.03 (1.36)	It is constructed from reinforced steel P = 4.40 (0.81) I = 3.86 (1.11) C = 3.71 (1.23)	Elbink	Gliptem
Object24	It is used to scrape inside hollow wooden logs P = 3.09 (1.38) I = 3.17 (1.27) C = 3.11 (1.32)	It is used to carry heavy wooden logs P = 2.83 (1.42) I = 2.71 (1.43) C = 2.80 (1.51)	It was once known as the “countryman’s friend” P = 3.77 (1.14) I = 2.69 (1.23) C = 2.89 (1.18)	It was once considered a status symbol P = 2.09 (1.09) I = 2.06 (1.26) C = 2.06 (1.08)	Tremba	Ganspet
Object25	It is used to scrape external drains P = 3.23 (1.29) I = 3.06 (1.11) C = 3.06 (1.26)	It is used to mark cuts of timber wood P 3.57 (1.09) I = 3.29 (1.20) C = 3.26 (1.31)	The blade is heavier than pure iron P = 2.94 (1.14) I = 2.60 (1.24) C = 2.74 (1.29)	The handle is made of animal bone P = 2.29 (1.36) I = 2.49 (1.38) C = 2.54 (1.29)	Gardat	Irbiff
Object26	This object scoops the marrow out of bone P = 3.89 (1.02) I = 3.63 (1.17) C = 3.54 (1.12)	This object measures exactly 1 g of sugar P = 3.34 (1.47) I = 3.31 (1.47) C = 3.00 (1.46)	This object is mainly used in Singapore P = 2.23 (1.29) I = 2.03 (1.12) C = 2.06 (1.11)	This object is mainly used in Alaska P = 2.20 (0.99) I = 2.11 (1.05) C = 2.26 (1.27)	Nomtaw	Carteep
Object27	It is used to groom pet hair P = 3.89 (1.21) I = 3.83 (1.22) C = 3.63 (1.19)	It is used to decorate iced cakes P = 2.89 (1.32) I = 2.86 (1.24) C = 2.80 (1.08)	This one is used by the Queen’s butler P = 3.51 (1.12) I = 3.17 (1.38) C = 2.97 (1.18)	This one can be found in “Harrods” of London P = 3.51 (1.31) I = 3.51 (1.36) C = 3.17 (1.22)	Hirpie	Tremba
Object28	It is used by children to make bubbles in the air P = 3.00 (1.28) I = 3.23 (1.46) C = 2.60 (1.29)	It is used by vets to hold open the mouths of horses P = 3.11 (1.21) I = 3.23 (1.24) C = 3.00 (1.31)	These are most commonly bought by Northern European vets P = 3.11 (1.32) I = 2.4 (1.24) C = 2.46 (1.01)	These are only manufactured in certain northern European countries P = 2.91 (1.27) I = 2.29 (1.05) C = 2.51 (1.17)	Millok	Tremba
Object29	It is used to loosen the mass around blocks in stone walls P = 2.80 (1.41) I = 2.57 (1.29) C = 3.00 (1.33)	It is squeezed in order to strengthen the muscles in the wrist P = 2.54 (1.17) I = 2.57 (1.33) C = 2.57 (1.38)	It comes with a lifetime guarantee and is considered to be unbreakable P = 3.00 (1.31) I = 2.57 (1.29) C = 2.77 (1.35)	It was once the most commonly exported object from Bangladesh P = 2.17 (1.12) I = 1.91 (1.04) C = 2.14 (1.12)	Pikstam	Oshkard
Object30	It is used to cut electrical cables P = 2.91 (1.42) I = 2.89 (1.32) C = 3.20 (1.45)	It is used to “crimp” metal P = 3.40 (1.09) I = 3.29 (1.30) C = 3.34 (1.24)	It once won a “badly designed object” award P = 3.03 (1.38) I = 2.86 (1.33) C = 2.37 (1.11)	It once won a prestigious design award P = 2.49 (1.22) I = 2.20 (1.18) C = 2.37 (1.24)	Conpi	Teeksmat
Object31	It is used in the washing and straining of root vegetables P = 2.23 (1.09) I = 1.80 (0.96) C = 2.31 (1.39)	It is used to wring the water out of clothing after washing P = 3.00 (1.33) I = 2.89 (1.32) C = 2.97 (1.44)	These objects were traditionally passed down from mother to daughter P = 2.80 (1.23) I = 2.37 (1.17) C = 2.46 (0.98)	These objects have only ever been found in the Southern Hemisphere P = 2.63 (1.37) I = 2.14 (1.17) C = 2.17 (1.20)	Gliptem	Sinfald
Object32	It is used to blow air on a fire P = 2.86 (1.43) I = 3.20 (1.30) C = 3.14 (1.33)	It is used to create holes in belts P 3.71 (1.23) I = 3.57 (1.22) C = 3.54 (1.34)	It is manufactured by a famous Yorkshire blacksmith P = 3.80 (1.11) I = 3.40 (1.22) C = 3.11 (1.23)	It is hung on the wall of a famous restaurant P = 3.40 (1.35) I = 3.17 (1.42) C = 2.89 (1.30)	Hantog	Teeksmat
Object33	It is used to mark locations P = 2.71 (1.34) I = 2.77 (1.35) C = 2.80 (1.35)	It is used to loosen soil P = 2.29 (1.05) I = 1.86 (1.03) C = 2.29 (1.34)	It is a ceremonial object P = 3.20 (1.21) I = 2.71 (1.20) C = 2.60 (1.029)	It is made of black opal P = 3.51 (1.17) I = 3.00 (1.19) C = 3.14 (1.17)	Jomtof	Arnco
Object34	It is used to stoke an open fire P = 3.97 (1.18) I = 3.83 (1.10) C = 3.71 (1.15)	It is used to test recently cooked meat P = 2.80 (1.39) I = 2.91 (1.38) C = 3.03 (1.27)	It was sold by traders in an African market P = 3.86 (0.94) I = 3.57 (0.98) C = 2.91 (1.04)	It was carried by ship captains in the 1800s P = 3.37 (1.19) I = 2.63 (1.19) C = 2.83 (1.27)	Dimpot	Daskon
Object35	It is used to exercise chest muscles P = 3.63 (1.52) I = 3.60 (1.50) C = 3.34 (1.30)	It is used to remove facial hair P = 2.03(1.34) I = 2.06 (1.39) C = 2.49 (1.69)	It is a top-seller on Amazon Japan P = 3.54 (1.34) I = 2.86 (1.38) C = 2.49 (1.40)	It was made famous by bloggers P = 2.60 (1.33) I = 2.20 (1.30) C = 2.03 (1.15)	Rogflan	Carteep
Object36	It is rolled over long-haired animals to remove parasites and foreign objects P = 2.74 (1.27) I = 2.66 (1.35) C = 2.66 (1.39)	It is rolled over pizza dough to create dimples and prevent over-rising during cooking P = 4.11 (1.16) I = 4.14 (1.00) C = 3.83 (1.25)	This one is a limited edition based on a design described in a futuristic novel P = 2.20 (0.93) I = 2.17 (1.20) C = 1.97 (0.98)	This one is solid silver and is owned by a famous American rap artist P = 2.29 (1.27) I = 1.97 (1.15) C = 1.94 (1.06)	Markap	Krandat
Object37	It is used to carve large blocks of ice P = 3.89 (1.25) I = 3.77 (1.11) C = 3.60 (1.33)	It is used to split large rocks in half P = 3.51 (1.15) I = 3.57 (1.14) C = 3.51 (1.17)	The seal on the handle is from the Danish king P = 3.23 (1.37) I = 3.00 (1.48) C = 2.77 (1.42)	The handle is made of the finest hickory wood P = 3.86 (0.94) I = 3.51 (1.07) C = 3.46 (1.36)	Ganspet	Hirpie
Object38	It is a medieval torture instrument P = 3.63 (1.29) I = 3.40 (1.38) C = 2.94 (1.39)	It is a sheep-shearing tool P = 4.14 (1.06) I = 4.01 (1.10) C = 3.94 (1.19)	It was discovered on a mountain top P = 3.29 (1.32) I = 3.00 (1.24) C = 2.71 (1.07)	It was discovered in a peat bog P = 3.51 (1.27) I = 3.20 (1.34) C = 3.11 (1.31)	Conpi	Kamstap
Object39	It is used to make marks in wet concrete P = 3.80 (1.13) I = 3.63 (1.19) C = 3.46 (1.12)	It is used to get stones out of horse’s hooves P = 3.80 (0.99) I = 3.54 (0.95) C = 3.54 (1.09)	This object was stolen by a gang of artefact thieves P = 3.31 (1.28) I = 2.91 (1.22) C = 2.71 (1.23)	This object was once used by Billy The Kid P = 2.46 (1.27) I = 1.91 (1.12) C = 1.91 (0.92)	Indayso	Saldol
Object40	It is held in the hand while punching P = 2.54 (1.36) I = 2.97 (1.34) C = 2.77 (1.29)	It is used to mash root vegetables P = 4.14 (1.03) I = 4.23 (1.03) C = 3.91 (1.12)	This object is banned in several countries P = 2.63 (1.24) I = 2.51 (1.25) C = 2.57 (1.33)	This object was blessed by the Pope P = 1.80 (1.13) I = 1.94 (1.28) C = 1.89 (1.21)	Bindo	Hingot
Object41	This is a grating/slicing device P = 3.51 (1.20) I = 3.83 (0.98) C = 3.40 (1.19)	This is a musical instrument P = 3.20 (1.53) I = 3.11 (1.37) C = 3.20 (1.55)	This was designed by prison inmates P = 2.94 (1.11) I = 2.66 (1.16) C = 2.49 (1.01)	This was designed by homeless people P = 2.54 (1.24) I = 2.09 (1.15) C = 1.91 (0.89)	Nimdo	Daskon
Object42	This is used by priests to sprinkle water on newborns P = 2.94 (1.30) I = 2.77 (1.33) C = 2.86 (1.33)	This is a Native Australian ceremonial tobacco pipe P = 3.43 (0.95) I = 3.26 (0.95) C = 3.31 (1.08)	This object is considered sacred by native Australians P = 3.23 (1.11) I = 2.63 (1.24) C = 2.37 (1.11)	This object is only ever used once every year P = 2.89 (1.41) I = 2.26 (1.20) C = 1.97 (1.15)	Vorbit	Gardat
Object43	This was used to cut chunks of sugar from larger blocks P = 3.63 (1.11) I = 3.49 (1.12) C = 3.43 (1.04)	This was used to remove staples from bundles of paper P = 2.80 (1.35) I = 2.80 (1.39) C = 3.06 (1.41)	This object was given as a gift to Abraham Lincoln P = 2.77 (1.29) I = 2.49 (1.25) C = 2.54 (1.34)	This was a popular object amongst the Victorian aristocracy P = 3.23 (1.09) I = 2.66 (1.19) C = 2.91 (1.15)	Remdip	Jomtoff
Object 44	Hot coals are placed in this object which is then used to warm a bed P = 4.54 (0.70) I = 4.51 (0.85) C = 4.29 (0.99)	Dried corn is placed in this object and is then “popped” over a flame P = 3.06 (1.47) I = 3.20 (1.49) C = 2.91 (1.58)	This object has been on permanent display at the Tower of London since 1666 P = 3.60 (1.31) I = 3.51 (1.42) C = 3.14 (1.50)	This object is thought to have survived the Great Fire of London in 1666 P = 3.37 (1.33) I = 3.00 (1.19) C = 2.80 (1.39)	Zamcon	Jafnog

* Unlike the functional facts, names were not always attached to the same objects and were randomly allocated during the experiments

## Footnotes

1 For example, https://www.reddit.com/r/whatisthisthing/

2 For the recognition scale, 1 = “I have never seen an object like this before”; 7 = “I recognise this object 100%”. For the scale asking about the function, 1 = “I have no idea what this object is used for”; 7 = “I know exactly what this object is used for”.

3 Two-tailed tests were used for the name-recognition comparisons because we did not have any theoretical reasons to make predictions in a specific direction. One-tailed tests would not change any of the conclusions.

4 This was because the program selected what was the “correct” answer in advance of participants’ selecting their answer. Whether they received confirmatory or disconfirmatory feedback was thus determined by the (pseudo)correctness of their response and was not counterbalanced.

5 To establish whether the unequal number of trials with confirmatory and disconfirmatory feedback was consequential, the following analyses were conducted. Firstly, a normality test was conducted on the distribution; Shapiro–Wilk statistic = .974, *df* = 51, *p* = .308. Skewness = .185, kurtosis =  − .196. This suggests that the distribution of deviations was normal and did not skew in favour of one condition. Additionally, the ANOVA was rerun after cases were weighted according to the relative proportion of trials in each feedback condition (e.g., a case in which there were 22 trials in each feedback condition was given a weighting of 0.5). After this weighting, the main results were unchanged from the original analysis; no main effect of feedback type, *F*(1,17) = .53, *p* = .478, η_p_^2^ = .03; main effect of information-type, *F*(1,17) = 11.44, *p* = .004, η_p_^2^ = .40; and a significant interaction, *F*(1,17) = 6.04, *p* = .025, η_p_^2^ = .262. These results suggest that the unequal numbers of trials in the feedback condition did not substantially impact upon the integrity of the results reported.

6 Plausibility: *M*_Functional_ = 3.32; *M*_Nonfunctional_ = 3.07; *t*(34) = 3.67, *p* < .001, Cohen’s *d* = .39. Concreteness: *M*_Functional_ = 3.20; *M*_Nonfunctional_ = 2.64; *t*(34) = 4.95, *p* < .001, Cohen’s *d* = .67. Imageability: *M*_Functional_ = 3.26; *M*_Nonfunctional_ = 2.73; *t*(34) = 5.91, *p* < .001, Cohen’s *d* = .53. All ratings were on a 1–5 scale.

7 Items 28F1, 31F2, 44F2, 24F1, 5F1, 20F2, 28F2, 7F1, 9F1, 2F1, 29N1, 20N1, 30N1, 23N1, 5N2, 12N1, 28N1, 4N2, 18N1, 15N2.

8 Items 28F2, 26F2, 6F1, 5F1, 7F1, 43F2, 14F1, 23F1, 20F2, 9F1, 9N2, 23N1, 32N1, 38N2, 33N2, 10N2, 44N1, 11N2, 7N1, 27N2.

9 Items 20F2, 10F1, 14F2, 2F1, 25F1, 41F2, 15F1, 6F1, 12F2, 23F1, 38N1, 44N2, 33N2, 4N2, 37N1, 5N1, 14N1, 27N1, 33N2, 38N2.
